# A Facile, Low-Cost Plasma Etching Method for Achieving Size Controlled Non-Close-Packed Monolayer Arrays of Polystyrene Nano-Spheres

**DOI:** 10.3390/nano9040605

**Published:** 2019-04-12

**Authors:** Yun Chen, Dachuang Shi, Yanhui Chen, Xun Chen, Jian Gao, Ni Zhao, Ching-Ping Wong

**Affiliations:** 1 State Key Laboratory of Precision Electronic Manufacturing Technology and Equipment, Guangdong University of Technology, Guangzhou 510006, China; chenyun@gdut.edu.cn (Y.C.); dachuang.shi@gmail.com (D.S.); gaojian@gdut.edu.cn (J.G.); 2 School of Engineering, The Chinese University of Hong Kong, Shatin 999077, Hong Kong, China; 3 Key Laboratory of Precision Microelectronic Manufacturing Technology & Equipment of Ministry of Education, Guangdong University of Technology, Guangzhou 510006, China; yanhuichen2019@outlook.com; 4 School of Materials Science and Engineering, Georgia Institute of Technology, Atlanta, GA 30332, USA

**Keywords:** plasma etching, non-close-packed monolayer array, ultra-high aspect ratio nanowire, Si nanowire, metal-assisted chemical etching

## Abstract

Monolayer nano-sphere arrays attract great research interest as they can be used as templates to fabricate various nano-structures. Plasma etching, and in particular high-frequency plasma etching, is the most commonly used method to obtain non-close-packed monolayer arrays. However, the method is still limited in terms of cost and efficiency. In this study, we demonstrate that a low frequency (40 kHz) plasma etching system can be used to fabricate non-close-packed monolayer arrays of polystyrene (PS) nano-spheres with smooth surfaces and that the etching rate is nearly doubled compared to that of the high-frequency systems. The study reveals that the low-frequency plasma etching process is dominated by a thermal evaporation etching mechanism, which is different from the atom-scale dissociation mechanism that underlines the high-frequency plasma etching. It is found that the polystyrene nano-sphere size can be precisely controlled by either adjusting the etching time or power. Through introducing oxygen as the assisting gas in the low frequency plasma etching system, we achieved a coalesced polystyrene nano-sphere array and used it as a template for metal-assisted chemical etching. We demonstrate that the method can significantly improve the aspect ratio of the silicon nanowires to over 200 due to the improved flexure rigidity.

## 1. Introduction

Monolayer nano-sphere arrays attract great interest in both academic research and industry development, as the array structure can be widely utilized in applications such as photovoltaics [1,2], biosensing [3] and micro/nano-structure fabrication [4]. By dip-coating [5,6], spin-coating [7], or air–water interface self-assembling [8,9,10,11], close-packed monolayer nano-sphere arrays can be facilely achieved on substrates like silicon wafers and glass panels, and used as templates for subsequent procedures. Although the close-packed monolayer arrays can meet plenty of demands, non-close-packed monolayer arrays are required by other special scenarios, e.g., fabrication of ordered holes [12], nanowires [13,14,15,16,17], complex microstructures [18], and so on.

In general, a non-close-packed monolayer array can be derived from a close-packed monolayer array through shrinking technologies such as chemical etching [19,20], heat treatment [21], plasma etching [22,23,24,25,26,27,28,29], and so on. Among these methods, plasma etching is the most commonly used method due to its operational simplicity [30] as well as its compatibility with a wide range of polymer materials, such as polystyrene (PS) [22], and polymethyl methacrylate (PMMA) [8]. However, the etching efficiency and the resultant array morphology (in both the array scale and individual nano-sphere scale) of the plasma etching method are still not satisfactory. Two main classes of plasma treatment, namely capacitively coupled plasma (CCP) and inductively coupled plasma (ICP), were thoroughly studied [22,24,25] and have been demonstrated to be capable of fabricating non-close-packed nano-sphere arrays with the desired size. The working frequency of the systems are usually high (up to 13.56 MHz) and an assisting gas such as oxygen or argon is required to achieve the desired plasma function [29]. A major limitation of the systems is that they tend to yield nano-spheres with very rough surfaces [8,23,24,25], which may cause failures in subsequent fabrication procedures. Moreover, the etching rate is severely limited to several nanometers per minute due to the working principle of high-frequency plasma etching, i.e., dissociating nano-spheres atom by atom [31].

In this study, we demonstrate that a low frequency (40 kHz) plasma etching system can be used to achieve non-close-packed monolayer arrays of polystyrene (PS) nano-spheres with smooth surfaces. Compared with the high-frequency plasma system, the etching rate can be doubled, in addition to the advantage of the low cost of the low-frequency plasma system. We show that the PS nano-sphere size can be precisely controlled by either adjusting the etching time or etching power. In addition, using the non-close-packed monolayer array of PS nano-spheres as a template for metal-assisted chemical etching, we have achieved silicon (Si) nanowires with a high aspect ratio of over 200.

## 2. Materials and Methods

Monocrystalline silicon wafers (Si, (100)-oriented, N-type, heavily doped, 4 inches in diameter, purchased from TurboTechnology Co., Ltd., Harbin, China) were rinsed in an ultrasonic bath with deionized water (DI water) and ethanol for 10 min, respectively, followed by drying in flowing N_2_ flow. Colloid containing PS nano-spheres (2.5% by weight, storing in water, 510 nm in diameter, Polysciences. Inc., US) was treated under the ultrasonic condition for 600 s. PS nano-spheres colloid was then dropped onto the Si wafer which was fixed on a spin-coater. The spin-coater worked at 100 rpm for 10 min so as to make a uniform colloid film on the wafer and subsequently at 1000 rpm for 1 minute to get rid of abundant colloid. After that, the Si wafer was placed still for 3 h for water evaporating. Finally, a hexagonally ordered close-packed array on the Si wafer surface was achieved. As-prepared Si wafer was diced into 10 × 10 mm^2^ for later experiments. These processes were all carried out in the cleaning room where temperature and humidity were strictly controlled to 23 °C and 50%, respectively.

Two plasma systems with different radio frequencies (RF) were used in this work. One was a Diener Low-Pressure Plasma System (ATTO, Diener electronic GmbH + Co. KG) with a working frequency of 40 kHz and maximum power of 200 W. The low-frequency plasma was generated by the capacitively coupled plasma (CCP) discharge. The cylindrical borosilicate chamber of this system was horizontally placed and two semi-arc electrodes were axis-symmetrically assembled on the outer wall of the chamber. The samples were placed in the center of the chamber, thus they were just between the ground electrode and biased electrode. The other plasma system was a Mobile Cubic Downstream Asher (ibss Group, Inc., Burlingame, USA) with a working frequency of 13.56 MHz and maximum power of 100 W. The plasma source, generated by the inductively coupled plasma (ICP) discharge, was connected to the cylindrical chamber by a pipe from the side wall. Thus, the samples were placed on neither the ground electrode nor the biased electrode. During the etching, oxygen (O_2_, purity of 99.999%) or argon (Ar, purity of 99.999%) was used as the assisting gas. It should be noted that unless it is declared that assisting gas was used, experiments were carried out with the residual gas after the vacuum was built up. Before etching, stabilization processes for the vacuum pump and the gas flow were carried out for 600 s to obtain a reproducible plasma atmosphere. The pressure inside the etching chamber was monitored by a Pirani gauge. When etching with the residential air, as no assisting gas was introduced, the working pressures were 0.1 mbar and 0.04 mbar for the 40 kHz plasma machine and 13.56 MHz plasma machine, respectively.

During the metal-assisted chemical etching process for fabricating silicon nanowire, Ti with 3 nm in thickness and Au with 30 nm in thickness were deposited on the plasma treated PS nano-sphere array by an electron beam evaporator (Lesker LAB18) at a rate of 3 nm/s. After the deposition, the samples were immersed in an etchant containing 10 mL hydrofluoric acid (HF, 49 wt.%, RHAWN), 2 mL hydrogen peroxide (H_2_O_2_, 30 wt.%, Aladdin) and 20 mL deionized water (DI water, 18.2 MΩ∙cm, produced by Milli-Q) for several minutes. The samples were then rinsed by plenty of DI water after etching and dried by N_2_ gas.

The characterizations of self-assembled PS nano-spheres on Si samples and Si nanowires were performed on scanning electron microscopes (SEM, SU8010 and SU8220, Hitachi). Image-Pro Plus (Version 6.0.0.260, Media Cybernetics, Inc, Rockville, USA.) was used to measure the diameter of the PS nano-spheres after etching. Polynomial curve fitting was conducted by Curve Fitting Toolbox 3.5.1 of MATLAB (R2015a, MathWorks, Inc.). Surface morphology analysis was performed on atomic force microscopic (AFM, Dimension FastScan, Bruker).

## 3. Results

**Figure 1:**
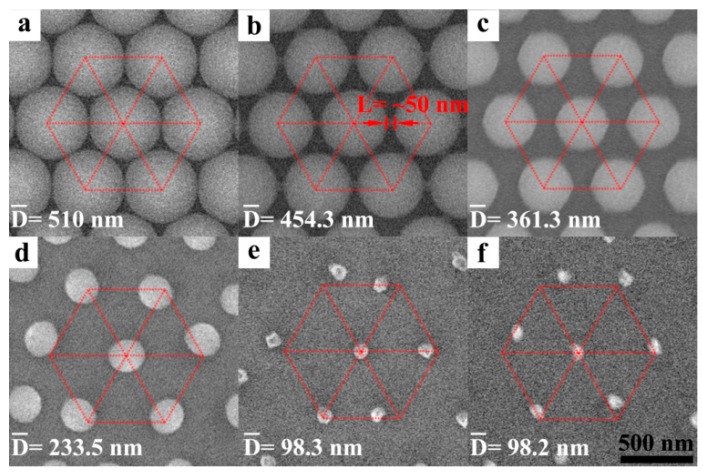
Scanning electron microscopes (SEM) images of polystyrene (PS) nano-spheres during the plasma etching. (**a**) Close-packed PS nano-sphere array before plasma etching. Images of the PS nano-spheres when they were etched for (**b**) 5 min, (**c**) 10 min, (**d**) 15min (**e**) 20 min, and (**f**) 25 min, respectively. The measurements were carried out by the best-fit circle tool of the Image-Pro Plus software and 10 nano-spheres were measured for each experiment to minimize random errors. The mean value was used to represent the diameter of nano-spheres. The hexagon in red demonstrates the original lattice of self-assembled PS nano-spheres. A bridge between the neighboring PS nano-spheres in (**b**) was marked in red.

Figure 1 shows the morphology evolution of the PS nano-spheres under the 40 kHz plasma etching (power of 100 W, without any assisting gas). Before etching, PS nano-spheres were closely packed into a monolayer, thus, there were only small gaps between the neighboring PS nano-spheres (Figure 1a). Once the plasma etching was applied, the PS nano-sphere surfaces were chemically dissociated and melted by the thermal energy which was provided by the plasma ions. When the plasma etching time was increased to 5 min, the molten polystyrene formed bridges with the neighboring PS spheres (Figure 1b). The bridges were about 50 nm in length. When the etching time was continued to increase to 10 min, the local temperature was increased correspondingly; thus, more materials were melted and then evaporated. In the meantime, the bridges were also chemically dissociated and melted, evaporated, and finally disappeared. Furthermore, the size of the PS nano-spheres was notably decreased; as a result, the PS nano-spheres were separated from each other and a non-close-packed array with PS spheres of ~ 361.3 nm in diameter was obtained (Figure 1c). When the etching time was further increased to 15 min, the PS nano-spheres were distorted a bit into an elliptical shape and some of them were dislocated from the original center positions due to the material evaporation (Figure 1d). After the etching time was increased to 20 min (Figure 1e) and 25 min (Figure 1f), the PS nano-spheres became irregular shaped dots and distributed somewhat randomly. However, the dot size no longer varied much with the etching time.

**Figure 2:**
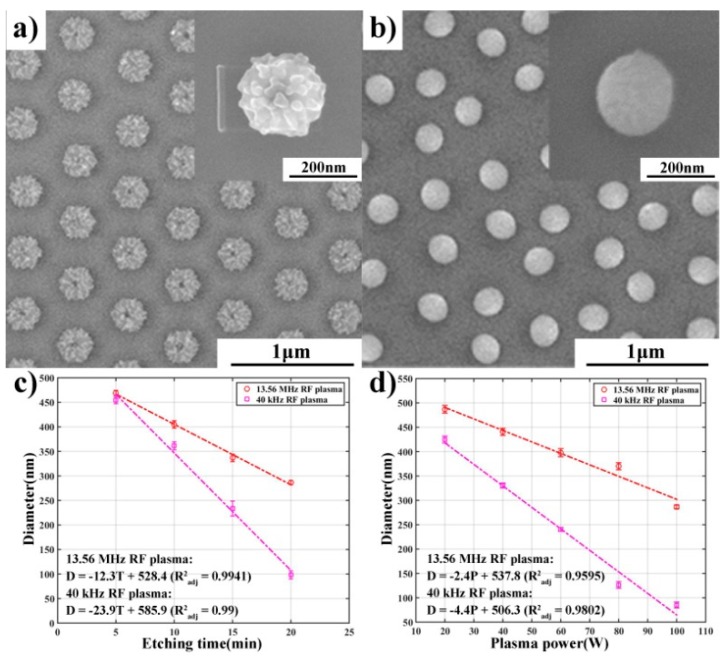
Comparison of the morphologies of PS nano-spheres etched by the plasma etching systems with different working frequencies. SEM images of the PS nano-spheres etched with the (**a**) 13.56 MHz and (**b**) 40 kHz plasma system, respectively. (**c**) PS nano-sphere size as a function of etching time. (**d**) PS nano-sphere size as a function of etching power. The etching time was kept at a constant of 20 min.

Figure 2 shows the comparison of the morphologies of the PS nano-spheres etched by the plasma systems operated at different working frequencies (the power was 100 W and no assisting gas was used). It can be seen that after etched by the 13.56 MHz plasma system for several minutes, the PS nano-spheres lost their original spherical shape and tended to adopt a hexagon shape. Furthermore, their surfaces became very rough (Figure 2a and Figure S1a, Ra ~5.5 nm). Similar results were reported by others [8,23,24,25], and only when doing the 13.56 MHz plasma etching under an extremely low etching temperature (−150 °C) can a more smooth and spherical shape nano-sphere array be achieved [25]. However, when the PS nano-spheres were etched by the 40 kHz plasma system, their spherical shapes were retained and the surfaces were much smoother (Figure 2b and Figure S1b, Ra ~1.2 nm). The sizes of the PS nano-spheres produced by different frequency etching were similar. Obviously, using a lower frequency plasma system and relaxing the requirement on the etching temperature is a more facile and economical approach.

To explore the controllability of the nano-sphere size during etching, we quantitatively studied the effect of etching time and etching power. Firstly, the relationships between the nano-sphere diameter and etching time under the two working frequencies were analyzed, as shown in Figure 2c. For all the experiments, the plasma power was kept at 100 W and no assisting gas was used. It can be noted that the size of the PS nano-spheres linearly decreased with the increased etching time for both working frequencies. Moreover, the size reduction of the PS nano-spheres under the 40 kHz plasma etching was much faster than that under the 13.56 MHz plasma etching. The etching rate (represented by the diameter reduction and calculated through one-order polynomial curve fitting) was 23.9 nm/min and 12.3 nm/min for the 40 kHz and 13.56 MHz plasma etching, respectively. It can be seen that the etching rate of the 40 kHz plasma systems was nearly doubled. Secondly, we studied the size reduction of the PS nano-spheres etched under different plasma power and with a constant etching time of 20 min, as shown in Figure 2d. It is noted that the PS nano-sphere size was reduced linearly with the increasing plasma power for both working frequencies, indicating that adjusting plasma power could be another effective approach to precisely control the size of the PS nano-spheres. Moreover, it is found that when using the 40 kHz plasma system, the PS nano-spheres were more sensitive to the plasma power. The etching rate of the 40 kHz plasma system (4.4 nm/W) was also nearly doubled compared with that of the 13.56 MHz plasma system (2.4 nm/W).

The above results demonstrated the superiority of the low-frequency plasma system for PS nano-sphere etching, as it can achieve a higher etching rate and obtain smoother sphere surfaces. The mechanism can be explained as follows. Unlike the high frequency (e.g., 13.56 MHz) plasma where the ion energies are more in the thermal range [32], in the low-frequency plasma (e.g., 40 kHz) the ions possess hyperthermal energies [33,34,35,36,37] and can effectively transfer their kinetic energy to the PS nano-spheres [38], resulting in a high temperature that enables the PS nano-spheres (the melting point of polystyrene was about 240 °C) to isotropically melt and vaporize at a high rate without chemical dissociation; additionally, the residual temperature anneals the PS nano-spheres and allows for formation of smooth surfaces due to the natural tendency of surface energy minimization. In contrast, the thermal ions from the high-frequency plasma are typically chemisorbed on the surfaces of the PS nano-spheres and then dissociate the PS nano-spheres atom by atom, as a result, the surfaces are rough and the material removal rate is low [39].

## 4. Discussion

### 4.1. Effects of Assisting Gas

**Figure 3:**
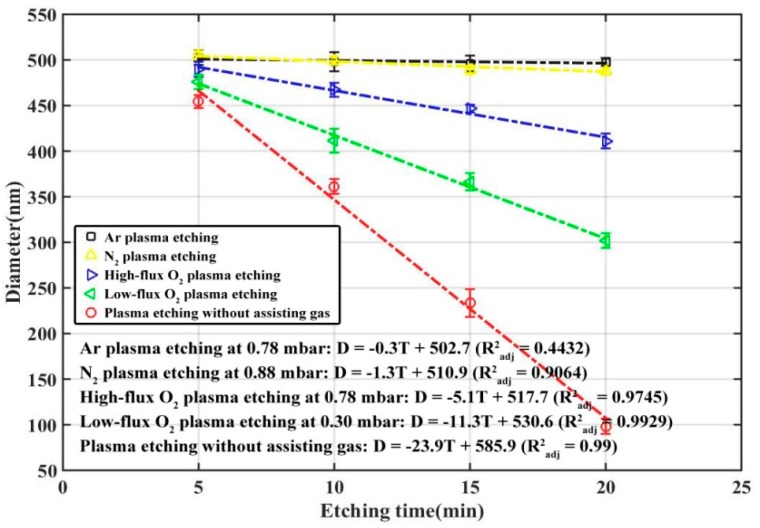
Size of the PS nano-spheres as a function of etching time for different gas types.

In the aforementioned experiments the plasma was generated from the residual air in the chamber. On the other hand, oxygen and argon are often introduced in CCP or ICP systems and used as the assisting gas to vary the etching rate. The effect of assisting gas on low-frequency plasma etching has rarely been studied. Figure 3 shows the effect of the gas type on the etching rate of PS nano-spheres. When argon was used in the plasma etching (the gas flux was strictly controlled to keep the chamber pressure stabilized at 0.78 mbar, referred to as the argon plasma etching), the etching rate was only 0.3 nm/min. It is almost the same trend when nitrogen was used as the assisting gas. When oxygen with the same flux was used instead (referred to as the high-flux oxygen plasma etching), the etching rate was increased to 5.1 nm/min. It is apparent that oxygen, as a reactive gas which reacts with carbon of PS to form volatile species, can enhance the etching rate compared with inert gases such as argon. On the other hand, we also note that when decreasing the oxygen flux to keep the chamber pressure at 0.30 mbar (referred to as the low-flux oxygen plasma etching), the etching rate can be remarkably increased to 11.3 nm/min. This may be due to too much oxygen flow bringing out the high-temperature ions and decreasing the temperature in the chamber. The hypothesis is also supported by the fact that the etching rate was largest (23.9 nm/min) when no assisting gas was used (referred to as plasma etching without assisting gas).

**Figure 4:**
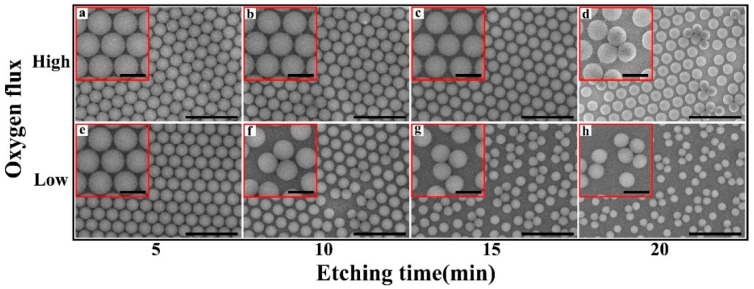
SEM images of the PS nano-spheres etched by the high-flux and low-flux oxygen plasma etching. PS nano-spheres etched by the high-flux oxygen plasma etching with an etching time of (**a**) 5 min, (**b**) 10 min, (**c**) 15 min, and (**d**) 20 min, respectively. PS nano-spheres etched by the low-flux oxygen plasma etching with an etching time of (**e**) 5 min, (**f**) 10 min, (**g**) 15 min, and (**h**) 20 min, respectively. The scale bar is 2 μm. The magnified image was inserted in each image. The scale bar in the magnified images is 500 nm. It should be noticed that coalesced nano-spheres firstly appeared in (**d**) and (**f**).

Figure 4 shows the effect of gas flux on the morphologies of the PS nano-spheres. When treated by the high-flux oxygen plasma etching (working pressure: 0.78 mbar), the PS nano-spheres gradually shrunk with the increased etching time (Figure 4a–d for 5, 10, 15 and 20 min etching, respectively), and narrow bridges linking the neighboring nano-spheres were initially established and then disappeared. After etching for 20 min, although most of the nano-spheres remained isolated, a few of them aggregated and coalesced nano-spheres appeared (Figure 4d). Note that this was not observed in the plasma etching without an assisting gas (Figure 1). Interestingly, when treated by low-flux oxygen plasma etching (working pressure: 0.30 mbar), the coalesced nano-spheres appeared much quicker, i.e., after only 10 min (Figure 4f). As the etching time was further increased, more and more nano-spheres coalesced, forming clusters containing two or more nano-spheres (Figure 4g,h). The results suggest that low-frequency oxygen plasma etching (40 kHz) could be used to form non-close-packed arrays containing coalesced nano-sphere clusters which can be applied as a template to fabricate special nanostructures. Moreover, the threshold of the diameter where the nano-spheres began to coalesce is estimated to about 411 nm (Figure 4d,f). The reason for such a size threshold can be understood as following: when the nano-spheres are large enough, i.e., when their diameter is above the threshold, they can be stabilized at their original positions perfectly, assisted by the bridges between them. Once the bridges disappear, the nano-spheres tend to move, following the vapor flow. As the nano-spheres continue to shrink, their contact area with the substrate is reduced and thus the contact force between the nano-spheres and the substrate is also weakened. As a result, more and more nano-spheres start to move and coalesce.

### 4.2. Fabricating Silicon Nanowires

The etchings that produced non-close-packed monolayer arrays with isolated or coalesced PS nano-spheres were used as templates to fabricate Si nanowires by the metal-assisted chemical etching (MACE) method [11,40,41,42].

**Figure 5:**
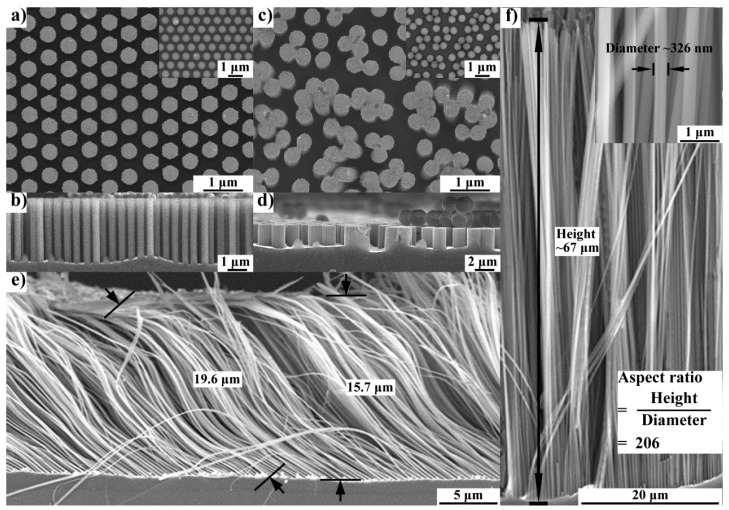
SEM images of Si nanowires fabricated by metal-assisted chemical etching (MACE). (**a**) Separated Si nanowires obtained using the separated PS nano-sphere array as the template (inserted). (**b**) Section view of the separated Si nanowires when they are short. (**c**) Coalesced Si nanowires obtained using the coalesced PS nano-sphere array as the template (inserted). (**d**) Section view of the coalesced Si nanowires when they are short. (**e**) Collapsed separated nanowires fabricated using the template in (**a**). (**f**) Ultra-long coalesced nanowires fabricated using the template in (**b**). An aspect ratio of 206 (~67 μm in length and ~326 nm in diameter) was achieved due to improved flexural rigidity.

When the template containing separated PS nano-spheres was used, we obtained ordered and uniform Si nanowires with a relatively short length (Figure 5a,b); when the Si nanowires became longer (i.e, >15 μm) as the etching time was increased, the Si nanowires collapsed due to the dramatically decreased flexural rigidity (Figure 5e). When the coalesced PS nano-sphere array was used as the template, coalesced Si nanowires were achieved accordingly (Figure 5c,d). Each coalesced Si nanowire consisted of two or more round nanowires, providing the nanowires a larger flexural rigidity. As a result, ordered Si nanowires with an aspect ratio over 200 were achieved (Figure 5f). The length of the coalesced Si nanowire can reach 67 μm. This method lights the way to fabricate ordered nanowires with ultra-high aspect ratios.

## 5. Conclusions

In summary, we explored the use of a 40 kHz plasma system for etching the monolayer arrays of PS nano-spheres. Compared with the high-frequency plasma system (up to 13.56 MHz), the 40 kHz plasma system can achieve a nearly doubled etching rate while maintaining the smooth surfaces of PS nano-spheres without the use of assisting gas. By adjusting the etching time and/or the plasma power, the size of the PS nano-spheres can be precisely controlled, providing a facile and economical approach for PS nano-sphere etching for industrial use. Through introducing oxygen as the assisting gas in the low frequency plasma system, non-close-packed monolayer arrays containing coalesced PS nano-spheres can be achieved. This study may provide a new strategy for industrial applications and insights for plasma etching. We further demonstrated that by using the coalesced PS nano-spheres as the template in the metal-assisted chemical etching of Si, we can achieve Si nanowires with an aspect ratio of over 200. This study can provide a new approach to fabricating ultra-high aspect ratio Si nanowires.

## Supplementary Materials

The following are available online at https://www.mdpi.com/2079-4991/9/4/605/s1, Figure S1: Surface morphology of individual nanosphere after plasma etching measured by AFM, Table S1: The details of etching conditions to obtain the results of Figure 1 and Figure 2, Table S2: Detailed data of Figure 2c, Table S3: Detailed data of Figure 2d, Table S4. Detailed data of Figure 3.
