# Supplementary material for: A Facile, Low-Cost Plasma Etching Method for Achieving Size Controlled Non-Close-Packed Monolayer Arrays of Polystyrene Nano-Spheres
Source: Nanomaterials (Basel). 2019 Apr 12;9(4):605. doi: 10.3390/nano9040605 (PMC6523458; doi:10.3390/nano9040605)
Supplement: Supplementary file 1 [file nanomaterials-09-00605-s001.pdf]

Supporting Information

A facile, low-cost plasma etching method for achieving size controlled non-close-packed monolayer arrays of polystyrene nano-spheres

Yun Chen[1,2], Dachuang Shi[1], Yanhui Chen[3], Xun Chen[1]*, Jian Gao[1], Ni Zhao[2]* and Ching-Ping Wong[3,4]*

1 State Key Laboratory of Precision Electronic Manufacturing Technology and Equipment, Guangdong University of Technology, Guangzhou, 510006, China
2 School of Engineering, The Chinese University of Hong Kong, Shatin, Hong Kong
3 Key Laboratory of Precision Microelectronic Manufacturing Technology & Equipment of Ministry of Education, Guangdong University of Technology, Guangzhou, 510006, China
4 School of Materials Science and Engineering, Georgia Institute of Technology, Atlanta, GA
* Correspondence: *xunchen@gdut.edu.cn(X.C);*nzhao@ee.cuhk.edu.hk(N.Z);*cpwong@cuhk.edu.hk(C.P.W)

Content:

Supplementary **Figure S1.** Surface morphology of individual nanosphere after plasma etching measured by AFM.

Supplementary **Table S1**. The details of etching conditions to obtain the results of Figure 1 & 2.

Supplementary **Table S2.** Detailed data of Figure 2(c).

Supplementary **Table S3**. Detailed data of Figure 2(d).

Supplementary **Table S4**. Detailed data of Figure 3.

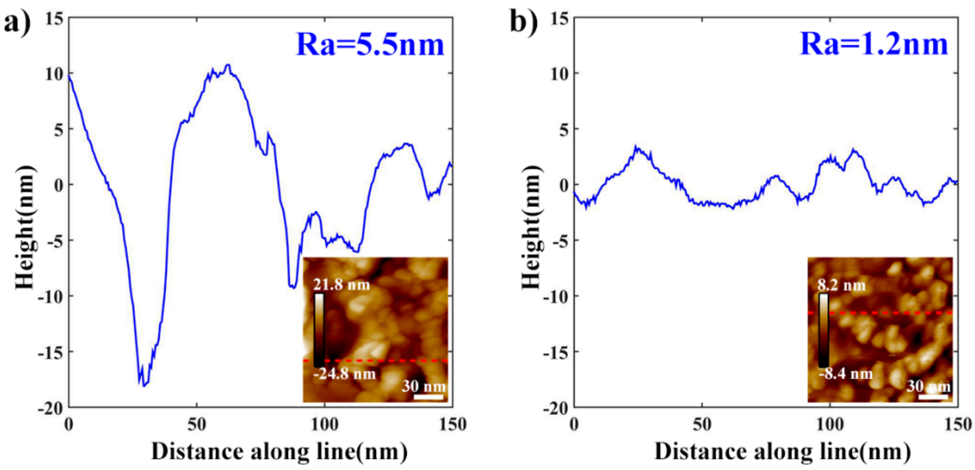

Figure S1. Surface morphology of individual nanosphere after plasma etching measured by AFM.

Section height plot of nanosphere etched by (a) 13.56 MHz plasma system and (b) 40 kHz plasma system, corresponding to the red dash line in the 2D image (insert).

Table S1. The details of etching conditions to obtain the results of Figure 1 & 2

Case	Gas	Power	Position
Figure 1	no assisting gas (residual air)	100 W	between the ground electrode and biased electrode.
Figure 2(a)	no assisting gas (residual air)	100 W	far away from both the electrodes
Figure 2(b)	no assisting gas (residual air)	100 W	between the ground electrode and biased electrode.
Figure 2(c)	no assisting gas (residual air)	100 W	between the ground electrode and biased electrode or far away from both the electrodes
Figure 2(d)	no assisting gas (residual air)	Varied power from 20W to 100W for different runs	far away from both the electrodes

Table S2. Detailed data of Figure 2(c)

13.56 MHz RF plasma (red line)				
Etching time (min)	5	10	15	20
Diameter (nm)	469.2	405.0	336.7	286.6
Standard deviation (nm)	5.2	7.7	7.7	4.1
40 kHz RF plasma (pink line)				
Etching time (min)	5	10	15	20
Diameter (nm)	454.3	361.3	233.5	98.3
Standard deviation (nm)	7.1	8.1	15.2	8.3

Note: The diameter of PS sphere on each sample was measured ten times to minimize random error.

Table S3. Detailed data of Figure 2(d)

13.56 kHz RF plasma (red line)					
Plasma power (W)	20	40	60	80	100
Diameter (nm)	487.0	440.6	397.9	370.0	286.6
Standard deviation (nm)	7.8	7.3	8.3	7.4	4.1
40 MHz RF plasma (red line)					
Plasma power (W)	20	40	60	80	100
Diameter (nm)	424.7	330.5	240.4	126.5	98.3
Standard deviation (nm)	7.1	5.1	4.0	7.0	8.3

Note: The diameter of PS sphere on each sample was measured ten times to minimize random error.

Table S4. Detailed data of Figure 3

Ar plasma etching				
Etching time(min)	5	10	15	20
Diameter(nm)	502.5	498.0	496.3	497.5
Standard deviation(nm)	8.0	10.5	8.5	4.7
N_2 plasma etching				
Etching time(min)	5	10	15	20
Diameter(nm)	505.1	499.2	489.1	487.6
Standard deviation(nm)	4.8	4.4	4.8	4.1
High-flux O_2 plasma etching				
Etching time(min)	5	10	15	20
Diameter(nm)	489.7	467.2	446.6	411.2
Standard deviation(nm)	8.4	7.7	4.2	8.2
Low-flux O_2 plasma etching				
Etching time(min)	5	10	15	20
Diameter(nm)	475.9	411.4	366.5	302.0
Standard deviation(nm)	7.9	13.1	9.4	8.0
Plasma etching without assisting gas				
Etching time(min)	5	10	15	20
Diameter(nm)	454.3	361.3	233.5	98.3
Standard deviation(nm)	7.1	8.1	15.2	8.3

Note: The diameter of PS sphere on each sample was measured ten times to minimize random error.
